# Brugada Syndrome: Warning of a Systemic Condition?

**DOI:** 10.3389/fcvm.2021.771349

**Published:** 2021-10-15

**Authors:** Sara D'Imperio, Michelle M. Monasky, Emanuele Micaglio, Giuseppe Ciconte, Luigi Anastasia, Carlo Pappone

**Affiliations:** ^1^Arrhythmology Department, Istituto di Ricovero e Cura a Carattere Scientifico (IRCCS) Policlinico San Donato, Milan, Italy; ^2^Faculty of Medicine and Surgery, University of Vita-Salute San Raffaele, Milan, Italy

**Keywords:** Brugada syndrome (BrS), sudden cardiac death (SCD), arrhythmia, genetics, epilepsy, ajmaline, thyroid, cancer

## Abstract

Brugada syndrome (BrS) is a hereditary disorder, characterized by a specific electrocardiogram pattern and highly related to an increased risk of sudden cardiac death. BrS has been associated with other cardiac and non-cardiac pathologies, probably because of protein expression shared by the heart and other tissue types. In fact, the most commonly found mutated gene in BrS, *SCN5A*, is expressed throughout nearly the entire body. Consistent with this, large meals and alcohol consumption can trigger arrhythmic events in patients with BrS, suggesting a role for organs involved in the digestive and metabolic pathways. Ajmaline, a drug used to diagnose BrS, can have side effects on non-cardiac tissues, such as the liver, further supporting the idea of a role for organs involved in the digestive and metabolic pathways in BrS. The BrS electrocardiogram (ECG) sign has been associated with neural, digestive, and metabolic pathways, and potential biomarkers for BrS have been found in the serum or plasma. Here, we review the known associations between BrS and various organ systems, and demonstrate support for the hypothesis that BrS is not only a cardiac disorder, but rather a systemic one that affects virtually the whole body. Any time that the BrS ECG sign is found, it should be considered not a single disease, but rather the final step in any number of pathways that ultimately threaten the patient's life. A multi-omics approach would be appropriate to study this syndrome, including genetics, epigenomics, transcriptomics, proteomics, metabolomics, lipidomics, and glycomics, resulting eventually in a biomarker for BrS and the ability to diagnose this syndrome using a minimally invasive blood test, avoiding the risk associated with ajmaline testing.

## Introduction

Brugada syndrome (BrS) is a hereditary disorder, highly related to an increased risk of sudden cardiac death (1), characterized by a type 1 (coved type) ST-segment elevation ≥2 mm followed by a negative T-wave in ≥1 of the right precordial leads V1 to V2 on the electrocardiogram (ECG) (2), which can occur spontaneously or after pharmacologically induced. The typical symptoms of BrS are syncope and cardiac arrest due to ventricular fibrillation (VF) or ventricular tachycardia (VT), in the absence of overt cardiac structural changes, typically between 30 and 50 years of age (3, 4). BrS has been associated with several other cardiac disorders, such as early repolarization syndrome (5), arrhythmogenic right ventricular cardiomyopathy/dysplasia (ARVC/D) (6–9), progressive cardiac conduction defect (Lenègre syndrome) (10, 11), LQTS (12–14), Wolff-Parkinson-White (15, 16), hypertrophic cardiomyopathy (17, 18), atrial flutter (19), and atrial fibrillation (20–23). In addition, BrS has also been associated with non-cardiac pathologies, such as epilepsy, thyroid disorders, cancer, skeletal muscle sodium channelopathies, laminopathies, and diabetes. One reason for this may be due to similar ion channel expression shared by the heart and other tissue types (24–27). In fact, the most commonly found mutated gene in BrS, *SCN5A* (28), is expressed throughout the entire body, with the largest protein expression levels found in the plasma, heart, and pancreatic juice (29). Consistent with this, large meals, alcohol, specific drugs, and fever can trigger arrhythmic events in patients with BrS, suggesting a role for organs involved in the digestive and metabolic (including mitochondrial) pathways (28, 30).

Drugs used to induced the type 1 BrS ECG pattern include Ajmaline, Flecainide, and Procainamide, which are often thought of as sodium channel blockers (4, 31, 32). Many centers prefer the use of Ajmaline because of its lower false-negative rate (33). However, several studies have highlighted the complex mechanism of Ajmaline, which does not act solely as a sodium channel blocker, but rather acts additionally on potassium and calcium channels (34, 35). Ajmaline can have side effects on non-cardiac tissues, such as the liver and mitochondria (36), further supporting the idea of a role for organs involved in the digestive and metabolic pathways in BrS.

In addition to cardiac, neural, digestive, and metabolic involvement, BrS may also affect other areas of the body. Potential biomarkers for BrS have been reportedly found in the serum or plasma of BrS patients (37, 38), although the conclusions are disputed (39). Indeed, a multi-omics approach would be appropriate to study this syndrome, including not only genetics, but also epigenomics, transcriptomics, proteomics, metabolomics, lipidomics, and glycomics (28). Here, we review the known associations between BrS and various systems and demonstrate support for the hypothesis that BrS is not only a cardiac disorder, but rather a systemic one that affects virtually the whole body.

## BrS Gene Candidates and Ajmaline Molecular Targets Expressed in Multiple Organ Systems

BrS was once viewed as a pure monogenic Mendelian disorder, caused by a single variant in a single gene. In some families, this may in fact still be the case. However, the view of BrS genetics has more recently evolved to recognize, in most cases, that BrS is an oligogenic syndrome, which can result from the combined effect of multiple variants, even in multiple genes, that occur in the same person (28, 40, 41). Much research is underway to determine what genes may be involved. Candidate genes (2, 28) commonly involve those encoding for sodium, potassium, and calcium channels (42–46), as well as, less commonly, sarcomeric and structural proteins (17, 18, 45, 47, 48) and mitochondrial genes (49) (Table 1).

**Table 1:** BrS candidate genes.

**BRUGADA SYNDROME CANDIDATE GENES**		
ABCC9	KCNE2	SCN2B
ACTC1	KCNE3	SCN3B
AKAP9	KCNE5/KCNE1L	SCN4B
ANK2	KCNH2	SCN5A
CACNA1C	KCNJ2	SCN10A
CACNA2D1	KCNJ5	SEMA3A
CACNB2	KCNJ8	SNTA1
CASQ2	KCNQ1	TMEM43
CAV3	RANGFR / MOG1	TNNI3
DSC2	MYBPC3	TNNT2
DSG2	MYH7	TPM1
DSP	MYL2	TRPM4
FLNC	MYL3	LMNA
GPD1L	PKP2	PLN
HCN4	PLN	CBL
JUP	NOS1AP	tRNA-Ala
KCND3	RYR2	tRNA-Gln
KCNE1	SCN1B	tRNA-Met

**Figure 1:**
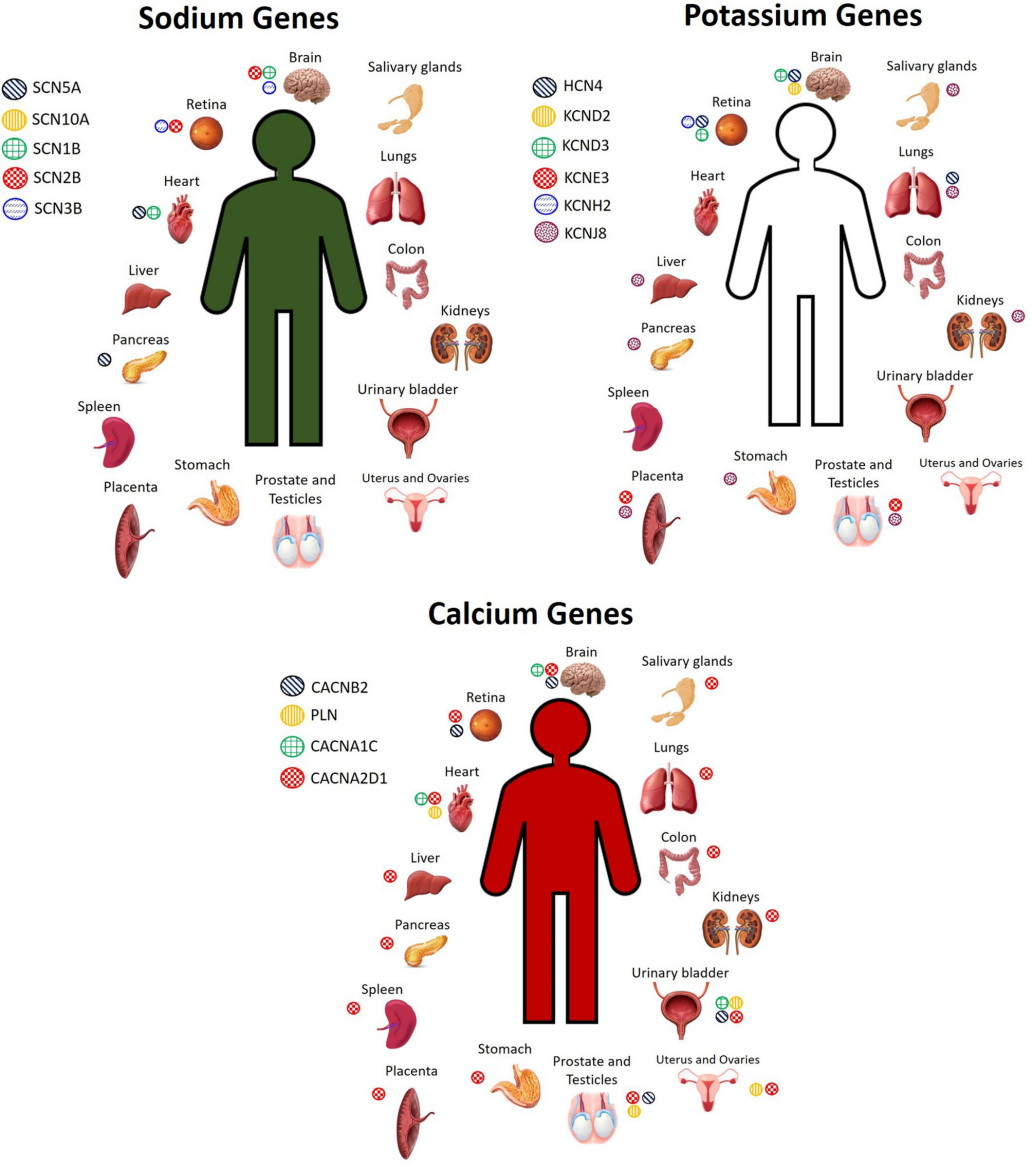
Cell and tissue types in which sodium, potassium, and calcium channel BrS candidate genes encode for high levels of protein expression. Not pictured: Sodium genes: *SCN5A*: plasma, pancreas juice. *SCN10A*: monocytes, neutrophil, B cells, T cells, skin. *SCN2B*: spinal cord, fetal heart, fetal brain. *SCN3B*: fetal brain. Potassium genes: *HCN4*: plasma, PBMCs, fetal heart. *KCND2*: fetal brain. *KCND3*: fetal brain, bones. *KCNJ8*: lymph node, bone marrow, stromal cells, nasopharynx, rectum, thyroid, adrenal, breast, gallbladder, skin, cervix, seminal vesicles Calcium genes: *CACNA2D1*: serum, plasma, PBMCs, lymph nodes, tonsil, bones, skin, bone marrow, esophagus, adipocytes, fetal brain, frontal cortex, cerebral cortex, cerebral spinal fluid, spinal cord, fetal heart, oral epithelial, cardia, fetal gut, rectum, thyroid, adrenal, gallbladder, urine, fetal ovary, fetal testis, seminal vesicles. *PLN*: tonsil, fetal heart, esophagus, urinary bladder, seminal vesicles. *CACNB2*: fetal brain. *CACNA1C*: frontal cortex, fetal heart, urinary bladder. *TRPM4:* Spinal cord, nasal respiratory epithelium, rectum, breast (29).

There are several candidate genes currently under investigation, reviewed elsewhere (2, 28, 40). Here, we do not seek to exhaustively list every single candidate gene that exists, but rather, we focus on a selected number of them to highlight the concept that these genes are expressed not only in the heart, but throughout the body, and thus mutations in any of these genes could have pathogenic effects not only on the heart, but also on other organ systems. The most widely screened BrS candidate genes encoding for sodium channels include *SCN5A, SCN10A, SCN1B, SCN2B*, and *SCN3B*, while genes encoding for potassium channels include *HCN4, KCND2, KCND3, KCNE3, KCNE5, KCNH2*, and *KCNJ8*, and genes encoding for calcium channels include *CACNA1C, CACNA2D1, CACNB2, PLN*, and *TRPM4* (2, 28, 40). These genes are expressed not only in the heart, but also throughout the rest of the body. Figure 1 shows the organs in which the protein level encoded by certain sodium, potassium, and calcium genes described in BrS is highly expressed. However, many of these genes encode for proteins that are expressed to a lesser extent in several other organs. Despite being expressed to a lesser extent, the low expression of these proteins in other cell types can influence disease expression and contribute to overlap pathologies. For more details on the organs and cell types in which these genes are expressed to a lower extent, see GeneCards (29).

**Figure 2:**
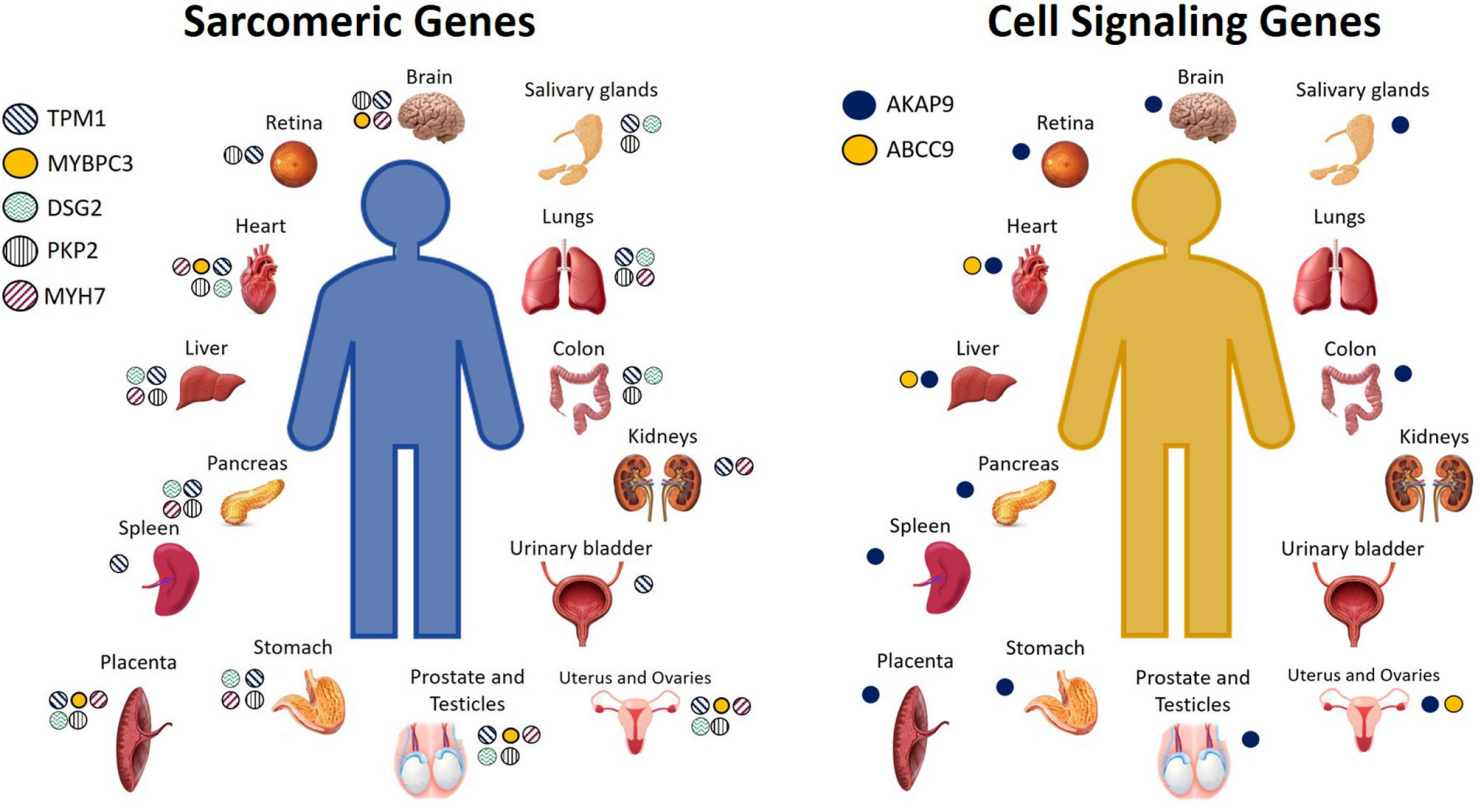
Cell and tissue types in which sarcomeric and cell signaling BrS candidate genes encode for high levels of protein expression. Not pictured: Sarcomeric genes: *TPM1*: Serum, plasma, monocytes, neutrophil, B lymphocytes, CD8 T cells, NK cells, PBMCs, platelets, lymph nodes, tonsils, bone marrow, fetal brain, spinal cord, fetal heart, bone, oral epithelial, nasopharynx, esophagus, cardia, gut fetal, rectum, fetal liver, adipocytes, amniocytes, thyroid, adrenal, breast, milk, gallbladder, urine, skin, fetal ovary, fetal testis, seminal vesicles. *MYBPC3*: fetal brain, fetal heart, oral epithelium, fetal liver, adrenal, fetal testis. *DSG2*: serum, plasma, platelets, tonsils, fetal heart, oral epithelia, esophagus, cardia, fetal gut, rectum, fetal liver, adipocytes, synovial fluid, amniocytes, thyroid, breast, gallbladder, urine, urinary bladder, skin, fetal ovary. *PKP2*: platelets, spinal cord, fetal heart, fetal gut, rectum, amniocytes, adrenal, breast, gallbladder, skin, fetal ovary, fetal testis. *MYH7*: platelets, tonsil, fetal brain, spinal cord, fetal heart, esophagus, rectum, fetal liver, adipocytes, fetal ovaries, fetal testis. Cell signaling genes: *AKAP9*: monocytes, PBMCs, platlets, lymph node, brain fetal, esophagus, cardia, rectum, fetal liver, adipocytes, amniocytes, adrenal, pancreatic juice, islet of Langherans, gallbladder, ovary fetal, testis fetal. *ABCC9*: plasma, bone marrow, heart fetal, fetal liver (29).

Calcium is the connection between excitation and contraction, and in fact, arrhythmias and sudden death can occur as a result of calcium mishandling, including altered calcium signaling resulting from sarcomeric mutations (50). Recent studies have suggested a possible involvement of sarcomeric mutations in BrS (17, 45, 51) that alter calcium signaling (18, 42). BrS sarcomeric gene candidates include *TPM1, MYBPC3, DSG2, PKP2, LMNA, MYH7, TTN*, and *GATA4* (17, 45, 47, 48, 52). In BrS, it has been proposed that loss of the action potential, and consequent reduced calcium channel current and cardiomyocyte calcium depletion, could result in wall motion abnormalities, dilation of the right ventricular outflow tract (RVOT) region, and reduced ejection fraction (EF) (31, 42, 53, 54), which have, in fact, been recently observed clinically in patients affected by BrS (55). BrS candidate genes encoding for proteins important in cell signaling include *AKAP9, ABCC9*, and *CBL* (40, 45, 56), and these, like the other BrS gene candidates, are expressed not only in the heart, but additionally throughout the rest of the body. The cell or tissue types in which these genes are highly expressed are shown in Figure 2. For a more comprehensive assessment of all the tissue types in which they are expressed even to a lesser extent, please see GeneCards (29).

Genes identified in BrS genetics research as candidate genes related to the mechanism of BrS are expressed throughout the human body (Figures 1, 2), and variants identified in any one of them could modify not only heart function, but the function of any tissue type in which they are expressed. To what extent is uncertain, and we summarize below studies which support the idea that we are only seeing the tip of the iceberg in what can be learned from BrS research, namely, the molecular connections between multiple pathologies throughout the body.

## Brugada Syndrome and the Neural System

An overlap between cardiac arrhythmia and epilepsy has been described, possibly because of common mutations in genes encoding for ion channels. Several studies point toward the possibility of co-expression of ion channels in both the heart and brain, leading to the manifestation of both cardiac arrhythmia and epilepsy (57–59). Many of these studies have identified gene mutations known to cause cardiac arrhythmia syndromes such as LQTS and BrS. For example, idiopathic epilepsy and LQTS may be linked by mutations in the potassium channel gene, *KCNQ1* (58). Also the sodium channel gene, *SCN5A*, usually associated with SCD and BrS (60–62), has been of particular interest to understand the effects of specific mutations (63–68) or even polymorphisms (69–73) in this gene, and their relation with BrS, and this gene has been linked to sudden unexplained death in epilepsy (SUDEP) (74). In fact, this gene has already been implicated in an overlap syndrome involving both epilepsy and BrS (24). Indeed, up to 18% of patients affected by epilepsy die of SUDEP (59). Unfortunately, autopsy performed on SUDEP patients does not reveal the cause of death nor any evidence related to pulmonary or cardiac pathology (75); however, SUDEP and sudden cardiac death due to cardiac arrhythmia share a few risk factors: age and sex (76). Furthermore, it has also been shown through EEG/ECG combined studies that patients with true epileptic seizures have a high prevalence (33–44%) of cardiac arrhythmias (58, 77–81), including, at least, LQTS, BrS, QT dispersion, sinus tachycardia, T-wave alternans, bradyarrhythmia, or asystole, sometimes through common genetic mutations (25–27, 82–88). Potential gene candidates for SUDEP include *FBN1, HCN1, SCN4A, EFHC1, CACNA1A, SCN11A*, and *SCN10A* (89). *SCN10A* is also a gene candidate for BrS (70, 90, 91), and patients with this syndrome and variants in this gene have been shown to have similar clinical presentations to patients with variants in the gene *SCN5A* (43).

Episodes of cardiac arrhythmia in patients affected by epilepsy may also be induced by antiepileptic drugs, such as carbamazepine and lamotrigine, which are known to target sodium voltage-gated channels (92–94). Carbamazepine, an anti-epileptic drug used also to treat schizophrenia, induced the BrS ECG pattern in a patient with schizophrenia (95). In a patient with epilepsy and intellectual disability, the drug Lamotrigine induced the BrS ECG pattern, possibly in conjunction with a genetic predisposition for cardiac arrhythmia due to a variant in the *SCN9A* gene, also possibly in association with a genetic variant in the gene *AKAP9* (96). The overlap between epilepsy and cardiac arrhythmias demonstrated in several studies suggest that patients with either of these conditions should be checked for the other. Many of the variants thought to be responsible for cardiac arrhythmias occur in genes that have a low expression in the brain. However, their expression is nevertheless present, and, as can be seen from the literature, very relevant. Thus, future studies certainly should investigate these associations.

## Brugada Syndrome and Circulating Electrolytes, Poisons, and Blood

An elevated ST segment, which looks identical to the ECG pattern used to diagnose BrS, has been described as a result of potassium or calcium imbalances in the blood. Often, this type of ECG pattern discovered as a result of electrolyte imbalances is described as a BrS “phenocopy.” However, so much is not known about the mechanism of BrS, and given the fact that BrS has historically been defined more for what it is *not* than for what it *is*, it may actually be premature to discount ST segment elevations occurring in the presence of electrolyte imbalances in the quest to unveil the mechanism of BrS (41). Several studies have described hypokalemia (97–99), hyperkalemia (100–104), or hypercalcemia (105–107) in association with the BrS ECG pattern, or in association with an elevated ST segment that appears identical to the BrS pattern. Diseases related to these conditions, such as Gitelman syndrome, which results in potassium depletion, usually attributed to the *SLC12A3* gene, can lend new candidate genes to the field of BrS (108). In patients with severe hyperkalemia, the ST segment elevation, even if described at the time as a “phenocopy,” was described in association with malignant arrhythmias secondary to resting potential depolarization, reduced sodium current availability, and fibrosis at the right ventricular outflow tract (102), signs which are now typically described also in association with “true BrS,” whatever that is. Hypercalcaemia can be responsible for the ST segment elevation, resembling the BrS ECG pattern, and ventricular fibrillation, secondary to calcium-dependent loss of sodium channel function (106). This mechanism too is consistent with current theories about the possible mechanisms of “true BrS.” Furthermore, hypercalcaemia is associated with hyperthyroidism (109), which is additionally implicated in the development of an ST segment elevation resembling the BrS ECG pattern.

In addition to electrolyte imbalances, abnormalities of the blood occurring even from blood transfusion can result in the BrS ECG pattern. The type 1 BrS ECG pattern appeared after cardiac iron overload after blood transfusion (110). Another patient developed the BrS ECG pattern during febrile neutropenia after undergoing high dose chemotherapy followed by autologous peripheral blood cell transplantation (ABSCT) for acute myeloid leukemia (111). These cases of apparent transmission of the BrS could provide further insight into the mechanisms of the development of the BrS ECG pattern.

Plasmic proteomic changes have been observed in patients with BrS, including increased levels of apolipoprotein E, prothrombin, vitronectin, complement-factor H, vitamin-D-binding protein, voltage-dependent anion-selective channel protein 3, and clusterin (37). Similarly, decreased protein levels were observed for alpha-1-antitrypsin, fibrinogen, and angiotensinogen, and post-translational modifications of antithrombin-III were observed (37).

An elevated ST segment identical to the BrS ECG pattern can also be elicited by environmental factors, which ultimately act on the heart. It has been established that metals could induce this pattern. Several studies reported the manifestation of the BrS ECG pattern after the consumption of metal phosphides, which are mainly found in rodenticide, insecticide, and fumigant, and they are used as suicidal agents. These substances, such as aluminum phosphide (ALP), zinc phosphide (ZnP), calcium phosphide, and magnesium phosphide, are very toxic because they release phosphine gas as soon as they interact with hydrochloric acid in the stomach. The phosphine gas is a non-competitive inhibitor of cytochrome c oxidase in the mitochondria, and the mechanism of toxicity includes the formation of highly reactive free hydroxyl radicals (112). It can result in myocardial toxicity and cardia arrhythmia, which eventually can lead to death (113). Moreover, the intoxication from metal phosphides can result in several ECG abnormalities, including ST segment elevation in leads V1-V3, like the BrS ECG pattern. For example, three different cases of patients who ingested rat poison containing aluminum phosphide manifested significant abnormalities, including the ST segment elevation in leads V1-V3 (114–116). Moreover, another case reported a patient who intoxicated himself with rodenticide, which contained ZnP. The ECG of the patient showed a type 1 BrS pattern, which then normalized after 24 h (117). Finally, a male patient, after the ingestion of 7.5 g of Ratol paste, which is another rodenticide, showed prolonged PR interval, prolonged QRS duration, and the BrS ECG pattern (118). The manifestation of the BrS ECG pattern was related to the yellow phosphorous (YP), which is a protoplasmic poison contained in the paste, and it can cause damage to the liver, kidney, pancreas, brain, and heart (119). These reports about metal poisoning eliciting a BrS ECG pattern are also consistent with the fact that metals, such as zinc and copper, can act as endogenous regulators of sodium, potassium, and calcium channels, including Na_V_1.5, the sodium channel most implicated in BrS, through mechanisms that could be important not only for the heart, but also for diseases such as Alzheimer's disease and epilepsy (120). Thus, metals could play an important clue in understanding the various ways in which the BrS ECG pattern can be developed.

Whether through genetics, electrolyte imbalance, blood transfusion, poisoning, or other factors, the ECG pattern exhibiting an elevated ST segment can be developed in a number of different ways. However, regardless of the many initiating factors, all these pathways ultimately converge into one: the BrS ECG pattern, and we must not lose sight of the fact that, whatever we want to call it—“true BrS” or “phenocopy”—all of these initiating factors have converged into the same life-threatening arrhythmogenesis pathway, manifesting as the BrS ECG pattern, and the patient is at increased risk of ventricular fibrillation and SCD.

## Brugada Syndrome and Thyroid Dysfunction

Several studies have identified and described the relationship between thyroid hormone imbalances and cardiovascular diseases (121). It is known that thyroid hormone receptors are expressed in many different cell types, including heart (122), and, therefore, systemic vascular resistance, cardiac contractility, blood pressure, myocardial oxygen consumption, and heart rhythm can be disrupted by both hypothyroidism and hyperthyroidism (123, 124). Indeed, both of these thyroid dysfunctions can lead to cardiovascular disorders, including atrial fibrillation and ventricular arrhythmia (121). These correlations were also confirmed by cardiac electrophysiological studies focused on the activity of multiple ion channel subunits, transporters, and exchangers (121, 125). Moreover, a possible overlap of hypothyroidism and BrS has been reported in three case reports. All cases showed three men affected by hypothyroidism with a BrS ECG pattern in leads V1-V3. The BrS ECG pattern disappeared in all patients when the thyroid function was normalized (126–128). Therefore, the normalization of the ECG was considered directly related with the restoration of thyroid hormone balance. Moreover, one of the three patients with hypothyroidism was also affected by liver dysfunction, which returned to normal after thyroid function went back to normal (128). Only one of the patients underwent genetic testing. This patient resulted positive for three common variants in the *SCN5A* gene (127). Furthermore, a possible overlap between hyperthyroidism and BrS has been reported. A male patient suffered a cardiac arrest, and after his resuscitation, his ECG showed a type 1 BrS pattern. The laboratory tests showed also low K^+^ levels, low TSH levels, and high FT4 levels. However, the BrS ECG pattern was only transient, it normalized after carbimazole administration, and the patient resulted negative to ajmaline and flecainide tests. Genetic testing was not performed (129). Moreover, another patient with Graves' hyperthyroidism developed ventricular fibrillation, and she was implanted with an ICD. An ECG that was performed after she was re-admitted for fever and pleural effusion exhibited the type 2 BrS ECG pattern, although the report is unclear whether she performed ECG at the time of the actual fever. Genetic testing revealed a likely pathogenic mutation in the *SCN5A* gene (130). Therefore, genetic studies could be helpful to better understand the link between the manifestation of the BrS ECG pattern and thyroid dysfunction.

## Brugada Syndrome and Cancers

The overexpression of voltage-gated sodium channels has been shown to be associated with metastatic behavior in a variety of human cancers, including breast cancer, prostate cancer, lung cancer, colorectal cancer, cervical cancer, lymphoma, and neuroblastoma (131, 132). Overexpression of the neonatal isoform of the voltage-gated sodium channel, Nav1.5 (nNav1.5), is associated with aggressive human breast cancer metastasis and patient death (131, 133, 134). Nav1.5 overexpression increases metastasis and invasiveness of breast cancer cells by altering H+ efflux and promoting epithelial-to-mesenchymal transition and the expression of cysteine cathepsin (132), possibly due to reduced expression of salt-inducible kinase 1 (SIK1) (135). Both neonatal and adult (aNav1.5) forms of Nav1.5 are expressed at the messenger RNA level in colorectal cancer (136) and the *SCN5A* gene encoding for the Nav1.5 channel protein is an important regulator of the colon cancer invasion network, involving genes that encompass Wnt signaling, cell migration, ectoderm development, response to biotic stimulus, steroid metabolic process, and cell cycle control (137). Therefore, in the field of cancer, drug treatments that aim to block Nav1.5-dependent inward currents are of interest. However, sodium channel blockers used to treat patients with cancer may exacerbate underlying predispositions for cardiac arrhythmias, as sodium channel blockers, especially those targeting the Nav1.5 channel, may provoke arrhythmias such as the BrS ECG pattern. In fact, drugs studied for possible use in cancer therapy, such as Ranolazine (138) or Tetrodotoxin (136), are known to act not only on cancer cells, but on other cells in the body. Ranolazine acts on cardiac sodium channels and sarcomeric proteins (139). Recent studies have implicated cardiomyocyte sarcomeric alterations as possible mechanisms of BrS (28). Tetrodotoxin, believed to be one of the most selective inhibitors of voltage-gated fast sodium channels, actually acts additionally on other channels, including cardiac L-type calcium channels (140). Calcium channels and their related proteins have been implicated in a number of cardiac pathologies, including catecholaminergic polymorphic ventricular tachycardia, congenital long QT syndrome, idiopathic ventricular fibrillation, hypertrophic cardiomyopathy, arrhythmogenic cardiomyopathy, dilated cardiomyopathy, heart failure, atrial fibrillation (141), and BrS (28, 42). Thus, the possible development of cardiac arrhythmias should be considered for any drugs used for cancer treatment.

## Brugada Syndrome, Testosterone, and Prostate Cancer

Although equally transmitted to both genders (142), BrS seems to be more predominant in males (143), possibly due to hormonal action, among other factors (42). Specifically, males with BrS have been described to have higher testosterone, serum sodium, potassium, and chloride levels compared to controls, after adjusting for age, exercise, stress, smoking, and medication for hypertension, diabetes, and hyperlipidemia (144). In the same study, males with BrS had significantly lower body-mass index and body fat percentage than controls (144). Thus, a link between high levels of plasma testosterone, BrS, and prostate cancer was suggested (145). Men with Brugada-like electrocardiogram patterns have a higher risk of prostate cancer independent of age, smoking habit, and radiation exposure, and thus, men with either a Brugada-like ECG or prostate cancer should be checked for the other (145). The BrS ECG pattern can disappear after surgical castration for prostate cancer (146). The hypothesis for hormonal involvement in the development of BrS is further supported by cases in which BrS signs and symptoms manifest only in adulthood in patients who tested ajmaline negative in childhood (147). Moreover, healthy men are known to have differences in the ECG, compared to healthy women, for example, shorter and faster repolarization and a shorter duration of QTc and JTc intervals (148), supporting the idea that ventricular repolarization can be disrupted by androgens. Furthermore, no sex-differences in repolarization are observed in neonates or in children before puberty, probably due to low levels of testosterone (149). Cases in which BrS is found in pre-adolescent children, including also infants or when it is expected to be the cause of spontaneous abortions, may be due to certain genetic factors found in those families. For example, certain *SCN5A* mutations are known to be so detrimental that they can lead to sudden infant death (150), or they have been suspected as the cause of spontaneous abortions (151). In these cases, testosterone may play less (if any) of a role. However, testosterone is certainly one of the many factors that may contribute to the development of the BrS ECG pattern in many patients. Thus, future studies should investigate further the relationship between testosterone levels and the development of both BrS and prostate cancer.

## Mitochondria and Brugada Syndrome

**Figure 3:**
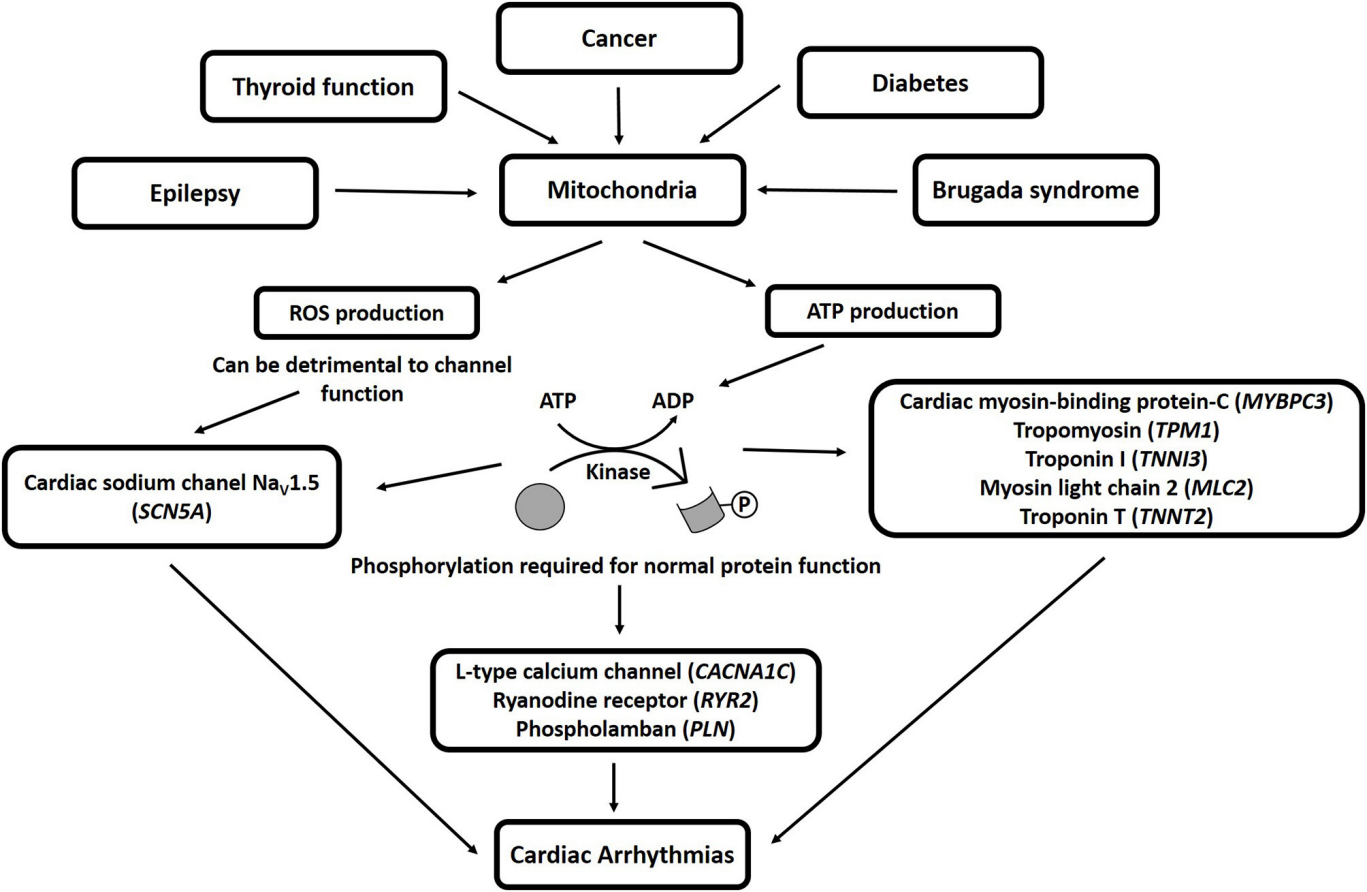
Mitochondrial products that can lead to arrhythmias. ROS, reactive oxygen species; ATP, adenosine triphosphate; ADP, adenosine diphosphate.

Mitochondria influence cellular physiology in a variety of ways (41), and are involved in many pathologies, including epilepsy (152, 153), thyroid function (154), cancer (155–158), diabetes (159, 160), and BrS (41), among others. Mitochondria produce adenosine triphosphate (ATP), which is required for protein phosphorylation, a process that is required for normal protein function (161). Protein phosphorylation is a normal regulatory process used by the cell to control the force-frequency relationship (the heart beats harder and relaxes faster to accommodate the need for higher heart rates) (162), the length-dependent relationship (Frank-Starling relationship, the heart beats harder when the muscle is stretched) (163), and other necessary intracellular communications (164–168). Proteins that are normally phosphorylated under certain conditions include several of those known to be important for arrhythmia development, including the sodium channel Nav1.5 (encoded by the *SCN5A* gene; important for BrS) (41), L-type calcium channels (encoded by the *CACNA1C* gene; important for BrS) (28), ryanodine receptors [encoded by the *RYR2* gene; important for catecholaminergic polymorphic ventricular tachycardia (169)], phospholamban [encoded by the *PLN* gene; important for arrhythmogenic cardiomyopathy and dilated cardiomyopathy (170)], cardiac myosin-binding protein-C (encoded by the *MYBPC3* gene; BrS candidate gene) (48), tropomyosin (encoded by the *TPM1* gene; BrS candidate gene) (28), and other sarcomeric proteins that regulate cardiac function (50, 163–166, 171), such as troponin I (encoded by the *TNNI3* gene), myosin light chain-2 (encoded by the *MLC2* gene), and troponin T (encoded by the *TNNT2* gene). Therefore, mitochondrial dysfunction that results in ATP depletion would alter the function of these proteins, possibly resulting in cardiac arrhythmias (50, 172–174). Furthermore, mitochondria are additionally responsible for the production of reactive oxygen species (ROS), and a mutation in the GPD1-L protein, encoded by an important BrS candidate gene (175), has been shown to promote ROS production, which could be detrimental to the sodium current (176). BrS has been associated with mutations in mitochondrial transfer RNA (tRNA) genes (49) and a specific mitochondrial DNA (mtDNA) allelic combination and a high number of mtDNA single nucleotide polymorphisms (SNPs) (177, 178). Thus, mitochondrial genes and function are important for BrS. Mitochondrial products that can lead to arrhythmias are shown in Figure 3.

## Brugada Syndrome and Diabetes

Ion channel dysfunction has been associated with diabetes mellitus (DM), leading to the development of a heart disorder called diabetic cardiomyopathy (179, 180), characterized by contractile dysfunction, abnormal cardiac electrical activity, mitochondrial dysfunction, arrhythmia, and sudden cardiac death (159, 160). Several patients affected by DM have been reported to exhibit the BrS ECG pattern, whether it was considered “true BrS” or a “BrS phenocopy” (104, 181–184), although a difference between “true BrS” and “BrS phenocopy” may not actually exist (41). A case report described a 16-year-old boy affected by DM and a mutation in the *GPD1L* gene who died suddenly during the night (185). The autopsy examination excluded hypoglycemia as the cause of death due to a full stomach and normal glucose levels in the vitreous humor. Indeed, the *GPD1L* gene is important for BrS, and may have been the cause of death (186). Variants in this gene have also been linked to sudden infant death syndrome (SIDS) (187). Therefore, a genetic link may exist between diabetes and BrS that could result in a phenotypic overlap. However, diabetes can also be caused by non-genetic factors, such as life style, food, persistent organic pollutants, and the gut ecosystem (188), and diabetes can cause fibrosis leading to diastolic heart failure with preserved ejection fraction, which may deteriorate into the development of arrhythmias and sudden death (189). Therefore, we cannot rule out that disease overlap is always caused by genetic factors, but instead the situation may be the evolution of one disease eventually resulting in the other, or there may be no mechanistic overlap at all in some patients (two different mutations, or a mutation and an environmental influence). In any case, an overlap exists between diabetes, cardiac arrhythmias, and sudden death that will be the subject of future investigations.

## Brugada Syndrome, Skeletal Muscle Sodium Channelopathies, and Laminopathies

The *SCN4A* gene encodes for the voltage-gated sodium channel Na_V_1.4, and its mutations are usually related to non-dystrophic myotonia, periodic paralysis, and myasthenic syndrome (190). This gene has also been implicated in overlap between BrS and skeletal muscle sodium channelopathies (122). Indeed, several patients with pathogenic mutations in the *SCN4A* gene and cardiac electrophysiological abnormalities have been described (191, 192). For example, a patient affected by non-dystrophic myotonia with a mutation in the *SCN4A* gene resulted positive to BrS with both flecainide and ajmaline challenges (192). Additionally, in a different study, the authors investigated whether BrS can be part of the clinical phenotype associated with *SCN4A* variants, and whether patients with BrS present with non-dystrophic myotonia or periodic paralysis and related gene mutations. Three patients from families with an *SCN4A*-associated non-dystrophic myotonia had also BrS. Also, the authors found a high prevalence of myotonic features in the families with BrS, involving other genes (191). Na_V_1.4 and Na_V_1.5 are both expressed in the skeletal muscle during embryogenesis (193), and they have 65% sequence identity (194). However, during the development, the relative Na_V_1.5 expression decreases, making Na_V_1.4 the most abundantly expressed sodium channel in skeletal muscle (193, 195). Taken together, patients with BrS or skeletal muscles channelopathies should be evaluated for the other.

Lamin A and C are intermediate filament proteins of the nuclear protein, and they are encoded by the *LMNA* gene. Diseases associated with the *LMNA* gene include Hutchinson-Gilford Progeria Syndrome, Emery-Dreifuss Muscular Dystrophy 2, Autosomal Dominant (29), and inherited cardiomyopathies, such as arrhythmogenic cardiomyopathy and dilated cardiomyopathy (125). A patient who experienced a cardiac arrest and manifested a BrS ECG pattern resulted positive for a variant in the *LMNA* gene, suggesting an overlap between BrS and laminopathies (125). Thus, this gene could be investigated in patients with BrS in future studies.

## Brugada Syndrome and Cardiac Disorders Overlap

There have been several excellent studies regarding the overlap between BrS and other cardiac pathologies, including early repolarization syndrome (5), arrhythmogenic right ventricular cardiomyopathy/dysplasia (ARVC/D) (6–9), progressive cardiac conduction defect (Lenègre syndrome) (10, 11), LQTS (12–14), Wolff-Parkinson-White (15, 16), hypertrophic cardiomyopathy (17, 18), atrial flutter (19), and atrial fibrillation (20–23). Therefore, whatever mechanisms that ultimately lead to the development of the BrS ECG pattern may also be involved in other pathways that result in other cardiac pathologies. These mechanisms may include genetic variants, fibrosis, altered calcium signaling, and anatomical substrate overlap.

BrS has been hypothesized to be like focal epicardial arrhythmogenic cardiomyopathy, and the final common pathway “reduced RVOT conduction reserve,” regardless of the genetics or non-genetic factors that may have been responsible. In that study, the patient's intrinsic RVOT conduction reserve was hypothesized to be age- and sex-dependent, with marginal reserves able to be exposed by the use of certain drugs or altered vagal tone (196).

### Genetic Overlap

The genetics of BrS remain elusive, as the most commonly found mutated gene, *SCN5A*, is only found to be mutated in a minority of patients. However, this gene is widely studied, and is the only one considered by some groups to have definitive evidence to be involved in BrS, while the involvement of the other genes is disputed (197). Since then, several other studies have been published supporting the role of other genes, although research is still underway to understand fully the genetic background of patients with BrS (44). The discovery of genes involved in BrS may be complicated by the possibility of BrS to be transmitted as a polygenic or oligogenic disease, making the data more complicated to interpret, and thus impeding the ability to reclassify these variants (40).

There is already a lot of evidence for genetic overlap between BrS and other cardiac pathologies. Overlap between BrS and LQTS type 3 has been described (198), even sharing the same mutation in the *SCN5A* gene as the likely molecular cause (13, 199). In fact, variants in the *SCN5A* gene have been described as causative for a number of other cardiac pathologies, such as ARVC/D, atrial standstill, atrial fibrillation, left ventricular non-compaction, dilated cardiomyopathy, LQTS, sick sinus syndrome, idiopathic ventricular fibrillation, and heart block (200, 201). In addition, several other genes associated with BrS are also associated with other cardiac pathologies, as previously reviewed elsewhere (202), such as *PKP2, DSC2, JUP, DSG2*, and *RYR2* in ARVC (108, 202), *SCN1B* and *CACNA1C* in LQTS, *CACNA1C, CACNA2D1*, and *CACNB2* in short QT syndrome, and *CACNA1C, CACNA2D1, CACNB2, HCN4*, and *KCNJ8* in early repolarization syndrome (202). Atrial fibrillation is associated with a number of genes that have been suspected in BrS, including SCN5A, SCN1B, SCN2B, SCN3B, SCN4B, SCN10A, ABCC9, HCN4, KCNQ1, KCNJ2, KCNJ5, KCNJ8, KCNE1, KCNE2, KCNE3, KCNE5, KCNH2, KCND3, RYR2, and CACNB2 (203). Therefore, genetics may play more of a role in the overlap of these pathologies than currently understood.

Disturbances of the connexome, the complex of structures of the intercalated disc that connect cardiomyocytes, could be involved in the mechanism of overlap between BrS and both arrhythmogenic cardiomyopathy and ARVC (9, 41). In addition to the overlap of several causative genes, the extracellular matrix and fibrosis may play a role in the overlap of several of these pathologies, as it is found to be present in BrS (204, 205), early repolarization syndrome (5), ARVC/D (206), Lenègre syndrome (207), LQTS (208–210), Wolff-Parkinson-White (211, 212), hypertrophic cardiomyopathy (213), atrial flutter and atrial fibrillation (214, 215). Thus, disturbances of the connexome and fibrosis can be important elements in the overlap of BrS with other cardiac pathologies.

### Arrhythmogenic Substrate and Anatomical Overlap

There may be an effect of anatomical overlap between BrS and other cardiac pathologies. For example, sarcomeric alterations, which are known to cause hypertrophic or dilated cardiomyopathy, are able to cause calcium mishandling that can lead to sudden death (216, 217), and so, over time, overlap may occur as the pathology progresses and affects other areas of the cell.

An arrhythmogenic substrate (AS) is consistently identified in all patients with BrS. This AS is commonly found in the RVOT of the right ventricle (RV) epicardium. Ajmaline administration reveals the full extent of the AS, which is useful for radiofrequency ablation, which results in ECG normalization and the patients are no longer inducible for VT/VF during electrophysiological study (EPS) (4, 218). The size of the AS has been independently associated with arrhythmia inducibility, with a substrate area of greater than 4 cm^2^ predicting patient inducibility to VT/VF during the EPS (219). The size of the AS can be altered not only by ajmaline, but by also a number of other factors, including genetics, temperature, anesthetics (220), and other factors known to trigger syncopic episodes or the BrS ECG sign. A larger AS is associated with reduced contractility and mechanical dysfunction (51). Patients harboring *SCN5A* variants exhibit more pronounced epicardial electrical abnormalities (60, 62), and the AS is comparable in patients with *SCN10A* variants (43). The procedure of epicardial mapping together with ajmaline challenge can be used to identify and ablate the AS in patients with other cardiomyopathies, thus preventing the recurrence of VT/VF in those patients as well (48).

## Future Directions

While BrS was described for decades as a simple monogenic disease originating only as a channelopathy, more recent studies have begun to recognize that actually BrS is an oligogenic disease with altered metabolics (28, 40). BrS has traditionally been described more for what it is *not* than for what it *is*, being described as a particular ECG pattern in the absence of certain criteria, but we believe that whenever the BrS ECG pattern is present, the sign should be taken for what it really is: a warning of increased risk of sudden death of the patient (41). It does not matter how the sign arose—from a channelopathy, a cardiomyopathy, an electrolyte imbalance, heavy meals, alcohol intake, or even poisoning—the BrS mechanism is a particular ECG warning of the increased risk of sudden death. Rather than being thought of as a single disease, it should be thought of as the final step in many pathways in many diseases or after exposure to certain environmental situations, that ultimately manifests as the BrS ECG pattern—a mechanism that can cause sudden death.

The BrS ECG sign does not always indicate solely cardiac dysfunction, but, in fact, this ECG sign could be the result of a larger systemic problem. Genetic mutations that are expressed in multiple cell types may contribute to overlap pathologies. We hypothesize that, with time, more overlap phenotypes will emerge in a host of other cell types, especially since many of the genes suspected to be involved in BrS are expressed throughout the body. Thus, future studies may focus on the effects of these genetic mutations in various cell and tissue types, identifying other problems for which the patients must be treated, as well as possible mechanisms for doing so. This could connect a whole magnitude of other pathologies to BrS, lending further evidence to the mechanism of BrS, arriving finally at better therapeutic options.

Ideally, where available, whole genome sequencing should be performed for the identification of new candidate genes. The effects of these specific genetic mutations in specific patients should then be assessed on a case-by-case basis to try to understand the effects of these mutations on other tissue types, in a move toward personalized medicine. Additionally, a multi-omics approach would be appropriate to study this syndrome, including not only genetics, but also epigenomics, transcriptomics, proteomics, metabolomics, lipidomics, and glycomics, resulting eventually in a biomarker for BrS and the ability to diagnose this syndrome using a minimally invasive blood test, avoiding the risk associated with ajmaline testing.

## Author Contributions

SD'I and MM: conceptualization, literature search, and writing—original draft preparation. CP: funding acquisition. SD'I, MM, and CP: writing—draft revision. SD'I, MM, EM, GC, LA, and CP: reviewed and provided comments. All authors have read and agreed to the published version of the manuscript.

## Funding

This study was partially supported by Ricerca Corrente funding from Italian Ministry of Health to IRCCS Policlinico San Donato.

## Conflict of Interest

The authors declare that the research was conducted in the absence of any commercial or financial relationships that could be construed as a potential conflict of interest.

## Publisher's Note

All claims expressed in this article are solely those of the authors and do not necessarily represent those of their affiliated organizations, or those of the publisher, the editors and the reviewers. Any product that may be evaluated in this article, or claim that may be made by its manufacturer, is not guaranteed or endorsed by the publisher.
